# Meniscal allograft transplantation in The Netherlands: long-term survival, patient-reported outcomes, and their association with preoperative complaints and interventions

**DOI:** 10.1007/s00167-020-06276-y

**Published:** 2020-09-26

**Authors:** Robert J. P. van der Wal, Marc J. Nieuwenhuijse, Reinier W. A. Spek, Bregje J. W. Thomassen, Ewoud R. A. van Arkel, Rob. G. H. H. Nelissen

**Affiliations:** 1 Department of Orthopaedics, Leiden University Medical Center, Albinusdreef 2, P.O. Box 9600, 2333 ZA Leiden, The Netherlands; 2 Department of Orthopaedic Surgery and Traumatology, Haaglanden Medical Center, The Hague, The Netherlands

**Keywords:** Meniscal allograft transplantation, Survival, Patient history, Patient-reported outcome, Satisfaction

## Abstract

**Purpose:**

Evaluation of survival of meniscal allograft transplantation (MAT) and postoperative patient-reported outcome (PRO), and their association with prior interventions of the knee.

**Methods:**

A prospective consecutive study of 109 consecutive patients who had an arthroscopic meniscal allograft transplantation (MAT) between 1999 and 2017 by a single surgeon. Patients were assessed with KOOS scores, preoperative and after a minimal follow-up of 2 years. Furthermore, two anchor questions (whether expectations were met and overall satisfaction, on a five-point Likert scale) were asked. Additionally, prior interventions to MAT were evaluated.

**Results:**

Prior to MAT, patients had undergone an average of 2.8 (range 1–14) of surgical procedures of the knee. Overall, mean allograft survival was 16.1 years (95% CI 14.8–17.5 years). Higher age at surgery was associated with lower MAT survival: hazard ratio for MAT failure was 1.19 per year increase (95% CI 1.04 to 1.36, *p *= 0.009). At 4.5 years (IQR, 2–9) of follow-up, all KOOS score were still improved compared to baseline. Age below 35 years, simultaneous anterior cruciate ligament reconstruction and number of knee surgeries before MAT were associated with lower KOOS scores. Overall patient expectations and overall satisfaction after MAT were not associated with preoperative patient characteristics nor with the number or kind of preoperative interventions.

**Conclusion:**

Meniscal allograft transplantation has a good overall survival with a clinically relevant improvement. Both meniscal allograft survival and PRO were associated with age. PRO was lower in patients younger than 35 years at time of MAT and meniscal allograft survival was worse in patients older than 50 years. PRO was associated with preoperative patient characteristics and number of surgical procedures prior to MAT. All patients reported improved postoperative satisfaction and met expectations after MAT, both independent of the preoperative history of knee interventions.

**Level of evidence:**

Level III.

**Trial registration** Medical ethical review board (METC) number: 17–104 (7 August 2017).

Dutch Trial Register (NTR) number: NTR6630 (4 July 2017).

**Electronic supplementary material:**

The online version of this article (10.1007/s00167-020-06276-y) contains supplementary material, which is available to authorized users.

## Introduction

Partial or total meniscectomy is often performed when damaged meniscal tissue cannot be repaired due to unfavorable conditions, type of meniscal tear, or when conservative treatment failed in the presence of a locking knee. Such a (partial) meniscectomy will alter the biomechanical and biological conditions in the knee joint [5]. This can lead to a painful meniscus deficient knee, also referred to as the *post*-*meniscectomy syndrome*, or eventually to symptomatic osteoarthritis [14]. In The Netherlands, about 18,000 to 36,000 arthroscopies for meniscal pathology are performed annually [21]. Subsequently, some of these patients develop a postmeniscectomy syndrome [17].

Currently, meniscal allograft transplantation is a widely accepted and recommended treatment option for patients with a postmeniscectomy syndrome [3, 24–26]. The first meniscal allograft transplantation (MAT) in The Netherlands was performed in 1989 [27], and until 1999, it was performed by an open procedure. Long-term results after open MAT show good clinical results [29]. Since 1999, MAT has been increasingly performed as an arthroscopically assisted procedure in The Netherlands, thus minimizing trauma to the knee joint [7]. Not only the surgical technique has been improved by the arthroscopic MAT, but also indications, patient selection, and postoperative rehabilitation have been improved during 30 years of clinical experience. Short- and long-term outcomes of both open and arthroscopic MAT have shown positive results in terms of pain relief and functional improvement [3, 25].

Interestingly, some patients with a poorer clinical outcome still report good levels of satisfaction about the MAT procedure. This suggests that patient satisfaction for MAT is likely multifactorial and poorly understood. Therefore, it is very relevant to obtain knowledge on preoperative patient characteristics which may influence not only clinical outcome, but also patient’s satisfaction. These factors can contribute to clinical decision-making on whether or not to perform an MAT.

Little is known on the associations of postoperative clinical results and patient satisfaction of MAT with preoperative history, symptoms, and prior conservative and surgical interventions. The latter may affect outcome after MAT. Publications reporting on patients’ history with respect to knee complaints prior to MAT, as well as interventions prior to MAT are scarce and often very concise [1, 18]. However, these factors may not only effect the knee joint and the lower extremity and the outcome of MAT, but also the patient’s overall functioning as reflected in the International Classification of Functioning (ICF) model of the World Health Organization (WHO) [30].

Therefore, as first aim of this study, the overall impact of clinical status and interventions prior to MAT on patient’s overall functioning after MAT using long-term patient-reported outcome measures (PROMs) was evaluated. As second aim, overall meniscal allograft survival at long-term follow-up was evaluated. Recently, minimal clinically important difference (MCID) and patient acceptable symptom states (PASS) for MAT were determined [11]; however, patients’ expectations and satisfaction about this procedure are still unknown and should potentially be considered as significant entities in the overall result of MAT. Thus, as our third aim, patient’s expectations and satisfaction about MAT were evaluated.

## Materials and methods

This study has been approved by the local medical ethical review board (METC Leiden-Den Haag-Delft, METC number: 17–104) and was registered in the Dutch Trial Register (NTR: NTR6630).

All 109 consecutive patients (111 meniscal allografts) with an arthroscopic-assisted MAT between November 1999 and January 2017 were evaluated. All surgeries were performed by a single experienced knee surgeon. Surgical technique is described in detail in earlier research [28, 29]. Patients eligible for MAT were 55 years and younger with disabling unicompartmental pain after a (sub)total meniscectomy with a stable knee joint or stabilized by concomitant anterior cruciate ligament reconstruction (ACLR) and normal knee alignment (5 degrees valgus–5 degrees varus).

Surgical technique: preoperative, size-matched, cryopreserved allografts were used. Needling of the remnant of the peripheral rim was performed to enhance MAT ingrowth. The allograft was fixed using 6–9 absorbable and non-absorbable sutures in the capsule, and no bone block was used. Until 2009, the posterior side of the allograft was attached to inside-out sutures. As of, 2009 all-inside meniscal sutures were used for posterior horn fixation. The middle part of the allograft was fixed with inside-out sutures. One tibia tunnel was used and the anterior horn was fixed to the tibia plateau using a suture anchor. Before 2009, an extra tibia tunnel was used for anterior horn fixation.

Rehabilitation started with 3 weeks of partial weight bearing (25%) with mobilization on crutches with limited flexion of 60°. After the first 3 weeks, partial weight bearing increased to 50% and knee flexion to 90°. From week 9 till 12, progressively loading was allowed and knee flexion to 120°. Between weeks 13 and 24, patients could progressively increase loading. If 80% of its former strength was reached, no restrictions were needed, except the advice to avoid high-impact activities and contact sports.

Baseline characteristics of the patient population are shown in Table 1. All patients had regular yearly clinical follow-up (including physical examination and assessment with PROMs). All patients were evaluated in 2019, with a minimum follow-up of 2 years. All patients gave written informed consent for this study.

**Table 1:** Baseline patient characteristics prior to meniscal allograft transplantation (MAT)

Number of patients with meniscal allograft	*n* = 109
Gender (female)—*n* (%)	65 (60)
Age (years)—median (IQR)	41 (29–51)
Medial compartment—*n* (%)	36 (33)
Bilateral	2 (2)
No. of concomitant ACL reconstructions—*n* (%)	16 (15%)
Median follow-up (months)—median (IQR)	54 (27–129)

Grade chondropathy^a^	Medial (*n* = 36)	Lateral (*n* = 69)
Grade 0	15	21
Grade 1	10	18
Grade 2	7	16
Grade 3	4	13
Grade 4	0	1

*ACL* anterior cruciate ligament, *IQR* interquartile range

^a^Worst grade of chondropathy on tibia and/or femur in treated compartment (Outerbridge scale)

At baseline, patients’ history of interventions prior to the meniscal transplantation as well as complaints of the knee and social impact of the knee complaints were evaluated at intake for the MAT procedure. For the current study, these data were collected from the medical records of the hospital’s electronic patient record system. Furthermore, at the final follow-up evaluation in 2019, all patients were asked to provide a complete overview of their medical history with respect to the knee prior and after MAT. Questionnaires were send out several times and multiple attempts were made to contact non-responders.

The Knee injury and Osteoarthritis Outcome Score (KOOS) [20] was used to evaluate the functional outcome. Health-related quality of life was assessed using EuroQol five-dimensional questionnaire (EQ-5D) [8]. To evaluate the postoperative patient opinion on the MAT procedure, two anchor questions with a five-point Likert scale were used (see appendix). The answers ‘to a reasonable degree’ (Likert scale 3) and higher were considered as a positive (i.e., satisfied on the postoperative result).

Preoperative data of one patient could not be retrieved. In 108 patients (99%), preoperative history of the knee was analyzed. At the final follow-up, 81 (74%) patients returned complete questionnaires. 28 patients were lost to follow-up after a median of 4 years [interquartile range (IQR) 2–13]. The two patients with bicompartmental MAT (1 lost to follow-up) were excluded for further analysis due to the small size of this group.

At final follow-up, 8 of 47 patients younger than 35 years were lost to follow-up (17%), 6 of 35 patients between 35 and 50 years were lost to follow-up (17%), and 5 of 27 patients older than 50 years at time of MAT were lost to follow-up (18%).

Meniscal allograft failure was defined as the removal of the complete allograft [with or without (unicompartmental) knee arthroplasty placement] as defined during the International Meniscus Reconstruction Experts Forum in 2015 [6].

### Statistical analysis

Data were tested for normality using Kolmogorov–Smirnov. Survival was assessed using Kaplan–Meier survival function and cox regression analysis (end-point: failure of meniscal allograft, see definition above). Continuous outcome measures were analyzed using a linear mixed model. This technique is recommended for the analysis of repeated measurements; it takes the correlation of values within subjects into account and deals effectively with missing values and loss-to-follow-up. Consequently, statistical inference can be based on the data of more patients then only those who completed the entire follow-up period [4, 16]. Continuous and ordinal variables are collapsed into ordinal variables or reduced in the number of categories if required for modelling purposes. For example, age is investigated in all models as a continuous variable, an ordinal categorized variable (younger than 35, 35–50, and older than 50), and a dichotomized variable (younger than 35, 35, and older). Regarding age, the dichotomized variable was chosen for modelling purpose and its clinical relevance [12]. Model assumptions were checked and models were adjusted accordingly.

In the mixed-model analysis, the following predictors were included: sex, age, side treated (left or right), compartment treated (medial or lateral), with or without concomitant anterior cruciate ligament reconstruction (ACLR), chondropathy grade two or more on the Outerbridge scale (yes or no) [13], and number of knee surgeries before MAT.

Postoperative ordinal and categorical outcome measures were analyzed using multiple linear, ordinal, and logistic regression models with appropriately collapsed outcome categories to obtain reliable estimates. Sex, age, side treated, compartment treated, with or without ACLR, chondropathy grade, and the number of knee surgeries before MAT were included as factors in the models.

IBM SPSS Statistics (version 25.0; SPSS Inc) was used for the statistical analyses.

## Results

### Patients’ history

Overall, 302 surgical interventions prior to MAT were performed in 108 patients (Table 2).

**Table 2:** Patients’ knee operations prior to and after MAT

Patients’ history^a^		
Number of prior surgical interventions	*n* = 302	Patients (*n* = 108)
1 operation		20 (19%)
2		38 (35%)
3–5		44 (40%)
6–14		6 (6%)

		Per patient (median, IQR)
Operations prior to MAT^a^		2 (2–3)
Partial meniscectomy		1 (1–2)
(Sub)total meniscectomy		0.5 (0–1)
ACL reconstruction		0 (0–0)
Meniscal repair		0 (0–0)
No further specified arthroscopy		0 (0–1)
Interval between (sub)total meniscectomy and MAT (months)		28 (13–68)

Operations after MAT	*n* = 48	patients (*n* = 29)^b^
Partial meniscectomy	12	8
Refixation of the graft	8	8
Resection of the graft	3	3
Total knee arthroplasty	8	8
Other reason (e.g., synovectomy)	17	16

*ACL* anterior cruciate ligament, *IQR* interquartile range

^a^Multiple responses (some patients had up to 14 prior procedures of which up to 7 partial meniscectomies)

^b^Some patients had multiple (different) operations after MAT

### Meniscal allograft survival

At the final follow-up, data on MAT survival data were available in 90 of the 109 patients (83%). In 29 patients (32%), a total of 48 operations were performed after the MAT (Table 2). Failure of the MAT occurred in 11 patients (10%); 2 medial (of 36, 6%) and 9 lateral (of 73, 12%) meniscal allografts failed after a mean of 8.0 years (range, 0.8–15.4 years). Eight of these patients (72.3%, or 7% of 109) had a conversion to total knee arthroplasty (TKA) at a median of 7.3 years (IQR, 5.2–11.9). The other 3 had a complete resection of the graft.

**Fig. 1:**
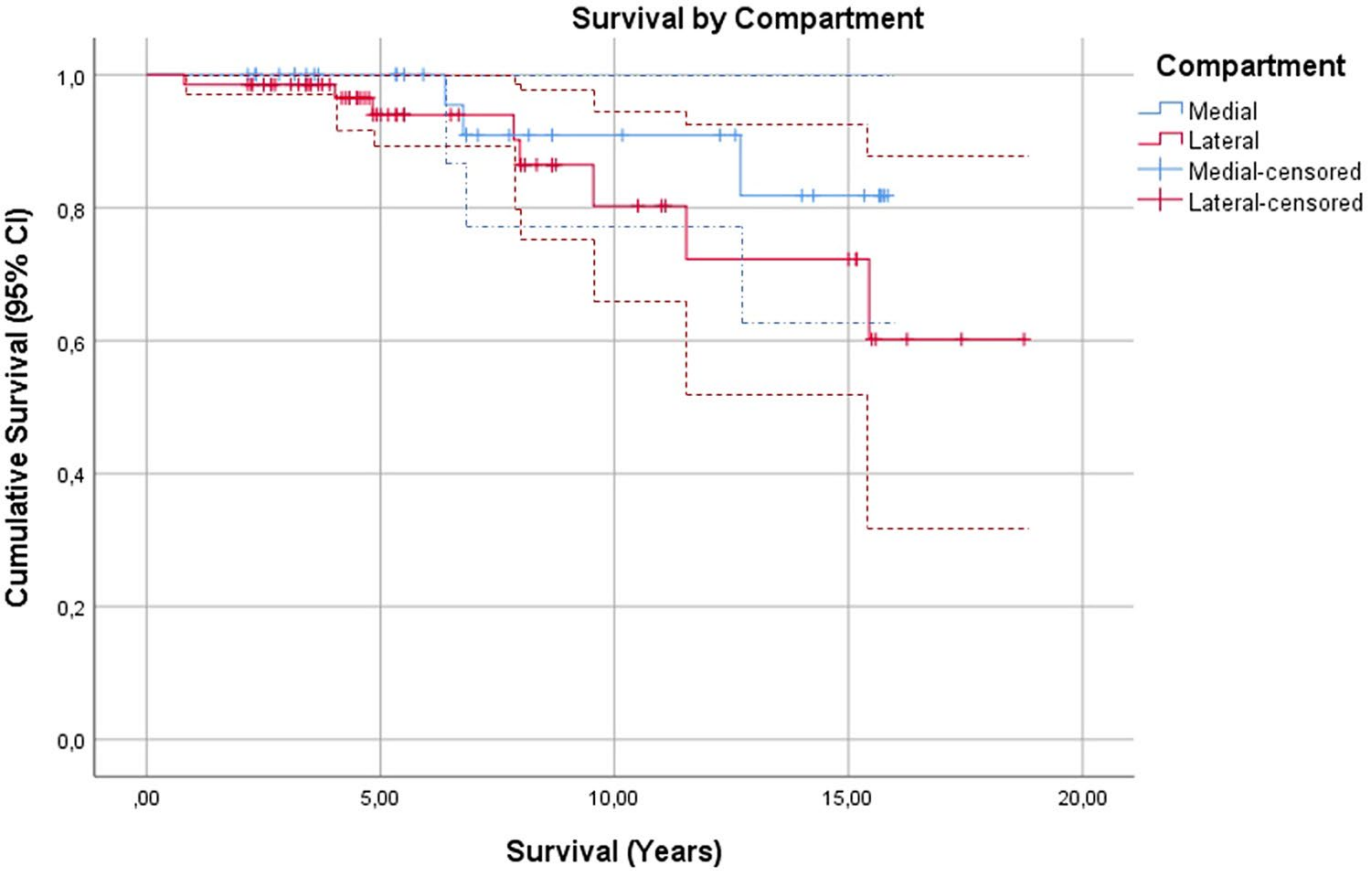
Survival by compartment (95% CI)

Mean survival time of the MAT was 16.1 years (95% CI 14.8–17.5). The mean survival of a medial and lateral MAT was comparable and did not significantly differ (Fig. 1).

**Fig. 2:**
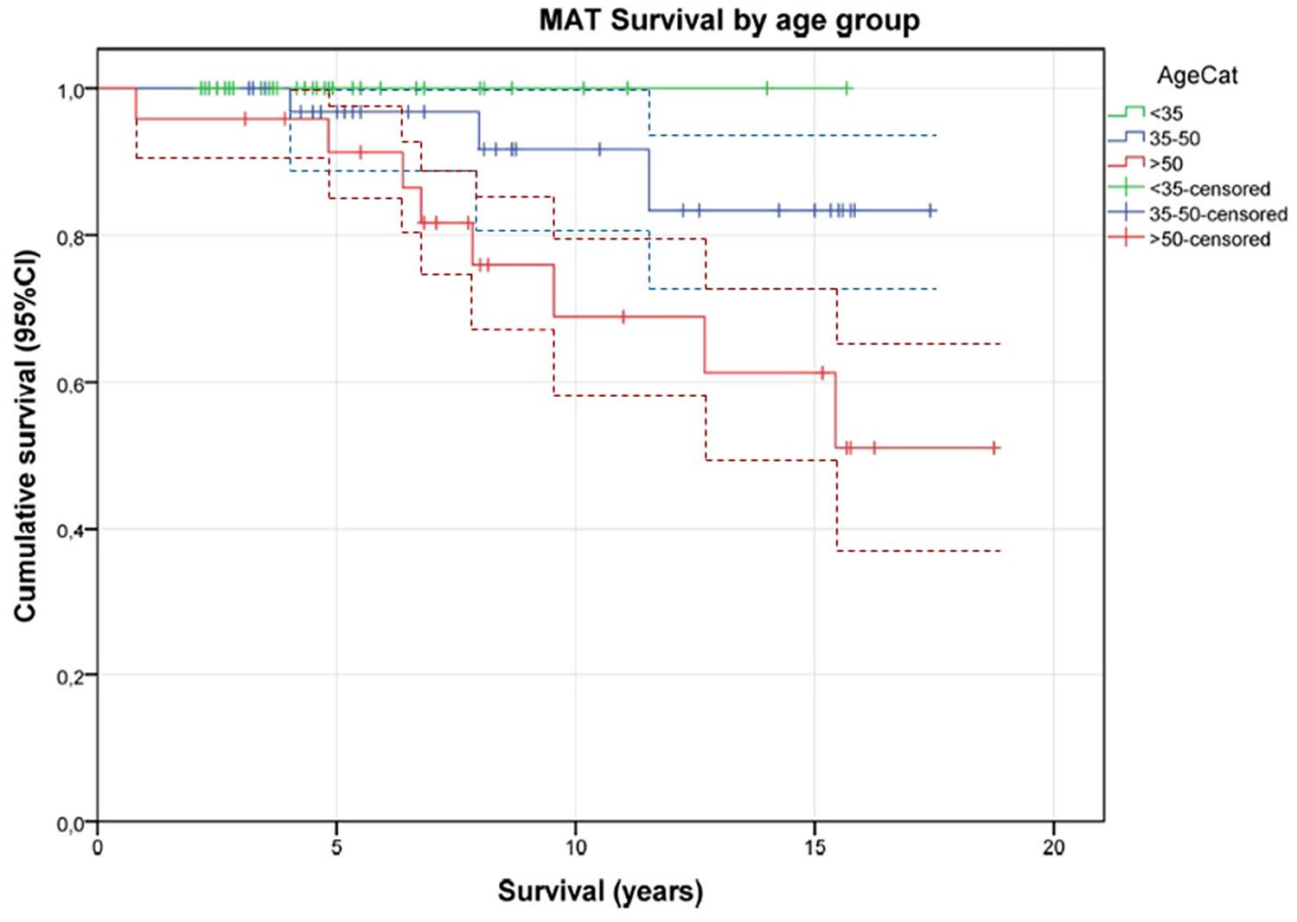
Survival by age (95% CI). *AgeCat* Age category

MAT survival was associated with age at baseline (Fig. 2, *p *= 0.010). In patients between 35 and 50 years, 3 of 29 (10%) had a failure of the MAT and in patients older than 50 years at time of MAT, 8 of 22 (30%) failed. Patient sex, compartment treated, procedure with or without ACLR, and intraoperative chondropathy grade two or higher (*χ*^2^ testing, *p* > 0.27) were not associated with survival of the MAT in an univariate analysis.

In a multivariable Cox regression analysis, patient age at baseline was associated with MAT survival: hazard ratio for MAT failure was 1.19 per increasing person year after the age of 35 years (95% C 1.04 to 1.36, *p *= 0.009). This corresponds to a 5.2 times higher risk of revision for every 10 year increase in age older than 35 years at time of MAT surgery.

### Patient-reported outcome

The median follow-up between MAT and final follow-up was 4.5 years (IQR, 2–9).

**Fig. 3:**
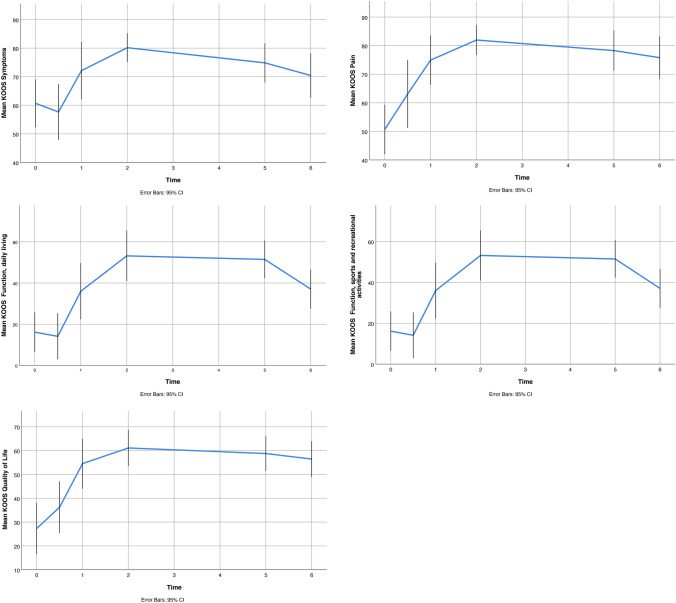
Mean KOOS scores at follow-up (mean/95% CI)

Overall, PROMs improved compared to the preoperative state and persisted during follow-up, except for a slight increase of symptoms after 5 years (Fig. 3). For all five domains, a minimal clinically important difference (MCID) was present at 1 year and at the last follow-up compared to baseline. The mean differences between scores at final follow-up compared to preoperative scores were significant for all five domains (Fig. 3).

With univariate analyses, age below 35 years, ACLR in the same session, and the number of knee surgeries before MAT were associated with lower improvement in different KOOS domain scores. Surgical side, knee compartment treated with MAT, and the degree intraarticular chondropathy were not significantly associated with the postoperative KOOS score (Table 3). Men experienced less improvement than women in the KOOS domain scores for pain (7.4 points, 95% CI 14.3–0.5, *p* = 0.037).

**Table 3:** Association between postoperative KOOS in relation to specified covariates (univariate analyses)

	KOOS symptoms	KOOS pain	KOOS function and daily living	KOOS sports and recreational activities	KOOS quality of life
Age	7.9 (95% CI 14.8–0.9) *p* = 0.028	5.6 (95% CI − 12.1 to 0.9) n.s	8.5 (95% CI 15.2–1.8) *p* = 0.015	14.0 (95% CI 24.7–3.6) *p* = 0.013	3.9 (95% CI − 13.0 to 5.4) n.s
Compartment (medial or lateral)	6.3 (95% CI − 1.5 to 14.2) n.s	1.8 (95% CI − 5.6 to 9.2) n.s	2.7 (95% CI − 10.6 to 5.2) n.s	2.3 (95% CI − 10.0 to 14.6) n.s	1.7 (95% CI − 9.2 to 12.5) n.s
Additional ACLR	16.3 (95% CI 5.1–27.6) *p* = 0.006	14.2 (95% CI 3.6–24.8) *p* = 0.01	2.3 (95% CI − 8.8 to 13.5) n.s	9.7 (95% CI − 6.5 to 25.9) n.s	8.8 (95% CI − 5.8 to 23.2) n.s
Side	0.3 (95% CI − 6.6 to 7.3) n.s	3.0 (95% CI − 3.6 to 9.5) n.s	3.3 (95% CI − 3.4 to 10.0 n.s	2.7 (95% CI − 13.3 to 7.9) n.s	2.8 (95% CI − 6.2 to 11.8) n.s
Number of surgeries before MAT	3.9 (95% CI 6.1–1.8) *p* = 0.001	3.9 (95% CI 6.0–1.8) *p* = 0.000	3.1 (95% CI 5.1–1.2) *p* = 0.002	2.6 (95% CI − 6.0 to 0.8) n.s	3.8 (95% CI 6.3–1.3) *p* = 0.003
Intraarticular chondropathy	0.7 (95% CI − 10.4 to 8.9) n.s	0.0 (95% CI − 9.1 to 9.2) n.s	7.2 (95% CI − 1.8 to 16.2) n.s	3.0 (95% CI − 10.7 to 16.6) n.s	3.1 (95% CI − 8.7 to 14.8) n.s

*95% CI* 95% confidence interval, *n.s* non-significant

At the final follow-up, overall mean postoperative quality-of-life score, EQ 5D, was 0.84 (95% CI 0.79 to 0.88). The mean patient-perceived health state on the EQ 5D 0-100 VAS was 78.5 points (95% CI 75.1 to 81.9), which was not associated with sex, age, surgical side, compartment, and concomitant procedure nor grade of chondropathy (*p* > 0.3).

Eighty patients (of 109, 73%) responded to the anchor questions; 4 questions were answered incomplete. The outcome for all 12 anchor questions was in the vast majority (range 71–93%) positive (scored 3 or higher on the Likert scale) (Table 4).

**Table 4:** Patient opinion on MAT procedure

	Not at all	Little	To a reasonable degree	Much	Very much	Negative/positive (positive %)
1. Expectations	3	4	15	42	15	7/72 (91%)
2. Confidence	7	14	18	31	8	21/57 (72%)
3. Social life	5	14	24	21	15	19/60 (76%)
4. Satisfaction body	7	15	25	25	7	22/57 (72%
5. Daily activity	5	12	14	35	14	17/63 (79%)
6. Work	9	14	18	25	14	23/57 (71%)
7. Solution to complaints	2	5	22	23	28	7/73 (91%)
8. Satisfaction	2	5	17	32	24	7/73 (91%)
9. Do it again	6	4	6	29	35	10/70 (88%)
10. Recommendation	1	5	12	29	33	6/74 (93%)
11. Physiotherapy	58	5	4	6	6	16/63 (79%)
12. Adjustments at work	28	22	17	9	4	13/67 (84%)

With regards to factors associated with patients, satisfaction multivariate analyses showed that patients of 35 years and older indicated that they were more willing to undergo the MAT again, compared to patients under 35 years (adjusted odds ratio 4.2, 95% CI 1.03–16.9, *p *= 0.04). Patients’ opinions on the outcome (e.g., expectation and satisfaction) of the MAT procedure were not associated with preoperative patient characteristics (e.g., grade of chondropathy), nor characteristics of the MAT procedure as such (e.g., ACL, and medial or lateral graft) (Table 5).

**Table 5:** Association between satisfaction of the treatment, fulfilment of the expectations, recommendation to undergo MAT again, and recommendation of procedure to other patients in relation to specified covariates

	Anchor questions			
	Preoperative expectation on MAT	Satisfaction on outcome MAT	Willingness to have MAT again	Recommendation to other patients
Sex	− 0.26 (95% CI 0.22–2.00, *p* = 0.50)	− 0.36 (95% CI − 0.24 to 2.00, n.s)	0.21 (95% CI − 0.36 to 4.18, n.s)	− 0.35 (95% CI − 0.21 to 2.34, n.s)
Side	0.54 (95% CI 0.60–4.66, *p* = 0.33)	0.51 (95% CI 0.60–4.67, n.s)	0.27 (95% CI − 0.40 to 4.32, n.s)	0.91 (95% CI− 0.75 to 8.16, n.s)
Compartment (medial or lateral)	0.55 (95% CI − 0.44 to 6.8, n.s)	0.72 (95% CI 0.53–8.07, n.s)	0.30 (95% CI − 0.28 to 6.54, n.s)	1.00 (95% CI − 0.50 to 14.84, n.s)
Additional ACLR	0.19 (95% CI − 0.20 to 7.3, n.s)	0.23 (95% CI − 0.21 to 7.47, n.s)	0.26 (95% CI − 0.19 to 8.87 n.s)	1.47 (95% CI − 0.60 to 31.59, n.s)
Number of surgeries before MAT	− 0.23 (95% CI − 0.59 to 1.1, n.s)	− 0.09 (95% CI − 0.60 to 1.40, n.s)	0.04 (95% CI − 0.77 to 1.41, n.s)	− 0.05 (95% CI − 0.71 to 1.27, n.s)
Intraarticular chondropathy	0.09 (95% CI − 0.72 to 1.68, *n.s*)	0.01 (95% CI − 0.68 to 1.51, n.s)	0.04 (95% CI − 0.66 to 1.64, n.s)	0.02 (95% CI − 0.63 to 1.53, n.s)
Age	0.50 (95% CI − 0.53 to 5.04, n.s)	0.13 (95% CI − 0.40 to 3.28, n.s)	1.43 (95% CI 1.03–16.90, *p* = 0.04)	0.09 (95% CI − 0.33–3.68, n.s)

Data expressed in B coefficient (standardised regression coefficient)

*95% CI* 95% confidence interval, *n.s* non-significant

## Discussion

The most important findings of this study were that both meniscal allograft survival and patient-reported outcome were associated with age; PROMs were lower in patients younger than 35 years and MAT failure rate was higher in patients older than 35 years. Patient-reported outcome was associated with (pre)operative characteristics: e.g., a higher number of knee surgeries before MAT and simultaneous ACLR were associated with lower PROMs. Nevertheless, all patients reported improved postoperative satisfaction and reported that preoperative expectations after MAT were met. The latter despite, an extensive preoperative history of knee interventions.

This is the first study evaluating all surgical procedures at the knee prior to MAT, using PROMs as well as MAT survival. Our results show a good survival of MAT, with an overall survival of 76% at 15 years. If MAT surgery was done at an age of 35 years and younger meniscal allograft graft survival was better. The risk of removal of the meniscal allograft increased with increasing age at time of MAT surgery. For every 10 years older age than 35 years at time of MAT surgery, the risk for meniscal allograft resection (with or without conversion to TKA) increased five times. Other patient and surgical characteristics, including sex, medial or lateral compartment, ACLR, or chondropathy, were not associated with graft survival.

The finding of a higher failure rate in older patients (> 35 years) is in concordance with others [12]. The biological vitality of the knee compartment in this age group might have a negative effect on graft ingrowth and subsequent graft degeneration. This might lead to a higher chance/likelihood of MAT failure. Furthermore, MAT survival is not only influenced by good graft ingrowth and functioning, but also on the decision whether or not to re-operate. This decision is likely age-dependent, as there are relatively few acceptable alternatives (e.g., unicompartmental or total knee arthroplasty) for these patients.

Regarding the high failure rate (30%) in patients > 50 years and the worse allograft survival compared to the younger age groups, it should be up for discussion to whether or not to perform MAT in these patients despite the positive results on satisfaction and expectations and despite the missing correlation between PROMs and grade of chondropathy in this patient group.

Twice as less failures were seen for medial MAT compared to a lateral MAT; however, in relation to allograft survival, there was no significant difference. This is probably due to the relatively small group of patients. This finding is supported by a meta-analysis by Bin et al. who also did not find a difference in survival between medial and lateral compartment MAT [2].

We found no inferior survival for concomitant ACLR and MAT, in concordance with the previous publications [2, 22]. Chondropathy grade three or higher was not associated with survival in our study. Where others [12] report the increased mechanical failure of MAT with advanced cartilage damage, we did not find this association. Even more, in our study, both patients with and without chondropathy benefit from MAT.

In the current study, all patients experienced a clinical improvement at 1 year until the final follow-up after MAT. Noteworthy is that despite a slight deterioration in clinical symptoms over time, the majority (88%) of patients were willing to undergo the MAT again, irrespective of eventual experienced MAT failure. Patients who were 35 years and younger at MAT surgery had lower PROMs, and reported consequently, to be more reluctant to undergo the same procedure again, for the same complaints. The worse KOOS scores in these younger patients probably reflect the higher physical demands and expectations on the effect of MAT surgery of these younger patients.

As mentioned earlier, this is the first study evaluating all surgical procedures at the knee prior to MAT, using PROMs as well as MAT survival. In our study, 82% of patients had two or more operations prior to their MAT. A higher number of knee surgeries before MAT had a negative association with postoperative knee score (KOOS). Multiple prior surgeries might lower the baseline value of patient-reported outcome, which also negatively influence the postoperative outcome. It could also be that the number of prior surgeries is related to the severity of injury or concomitant injuries prior to MAT, influencing the postoperative outcomes.

This is also the first study to evaluating patients’ expectations and satisfaction of MAT, taking into account prior procedures to the knee. For general meniscal surgery, it has been reported that patients expect fast recovery and a high level of participation in leisure activities [15]. However, in this study, less than half of the patients were able to participate at the same competition level as they expected preoperatively. Even more, less than 50% was satisfied with their knee function 3 months after meniscal surgery [15]. Although our MAT cohort with multiple surgical interventions at their knee was difficult to compare with a general meniscal lesion cohort, it was interesting to see that the MAT cohort showed more favorable results. In the current study, 91% of the patients met their preoperative expectations and 67% of patients were satisfied with the postoperative result. The satisfaction after MAT was confirmed by the large number of patients who would recommend the operation to patients with the same problem and who would undergo the same procedure again. Concordantly, Searle et al. also found a high number of patients (32 out of 43 patients, 74%) reporting that they would undergo MAT again [23].

Recently, Liu et al. determined the minimal clinically important difference (MCID) and patient acceptable symptom states (PASS) for MAT [11]. We did not establish PASS or MCID in our population, but positive answers to the questions about patients’ opinion regarding MAT ranged from 71 to 93%, with 91% in particular regarding patients satisfaction after MAT. We are aware that PASS thresholds can be patient population specific [11] and can alter in follow-up time [9, 10]. Nevertheless, compared to baseline level, all KOOS scores were above both the MCID and the PASS as given by Lui et al. [11] at any follow-up moment in our population. This confirms the patient-reported success of MAT in our cohort.

There are some limitations when interpreting the results of this study. First, 27% of patients were lost to follow-up, which could have led to selection bias. Although, a 60% threshold seems enough for an acceptable frequency of response [19]. Second, the wide range in follow-up since the MAT procedure might have an effect the patient’s opinion on the surgical procedure (recall bias). However, since we used anchor questions at follow-up, our results are informative on the patients’ perception of MAT.

Despite these limitations, the results of the present study could be used for a better expectation management in clinical practice. Based on patient characteristics (e.g., number of prior operations or patient’s age) expectations of a patient on the effect of an MAT can be better managed during the preoperative consultation prior to an MAT. Patients will be better informed during a shared decision-making process on outcome and MAT survival.

## Conclusion

Our results show a good overall survival of MAT, even in young patients, high patient-reported outcomes, and successful fulfilling of patient expectations. This makes MAT a good option a good joint preserving option, leaving other conservative and surgical options open. On the other hand, a higher number of previous procedures before MAT, simultaneous ACLR, and younger ages are associated with inferior patient-reported outcomes. These factors should be taken into account with clinical decision-making and expectation management with regards to MAT.

## Electronic supplementary material

Below is the link to the electronic supplementary material.

Supplementary material 1 (DOCX 13 kb)

Supplementary material 2 (DOCX 13 kb)
