# Supplementary material for: Meniscal allograft transplantation in The Netherlands: long-term survival, patient-reported outcomes, and their association with preoperative complaints and interventions
Source: Knee Surg Sports Traumatol Arthrosc. 2020 Sep 26;28(11):3551–60. doi: 10.1007/s00167-020-06276-y (PMC7591451; doi:10.1007/s00167-020-06276-y)
Supplement: Supplementary file 1 — Supplementary material 1 (DOCX 13 kb) [file 167_2020_6276_MOESM1_ESM.docx]

**Appendix I**

Questions on satisfaction (in Dutch)

|  | Helemaal niet | Nauwelijks | In redelijke mate | In hoge mate | In zeer hoge mate |
| --- | --- | --- | --- | --- | --- |
| 1. Komen de resultaten van de operatie overeen met uw verwachtingen hiervan? | 1 | 2 | 3 | 4 | 5 |
| 2. Heeft de operatie u zelfverzekerder gemaakt? | 1 | 2 | 3 | 4 | 5 |
| 3. Heeft de operatie een positief effect gehad op uw sociale leven? | 1 | 2 | 3 | 4 | 5 |
| 4. Bent u na de operatie meer tevreden over uw eigen lichaam? | 1 | 2 | 3 | 4 | 5 |
| 5. Gaan uw dagelijkse bezigheden u beter af dan voor de operatie? | 1 | 2 | 3 | 4 | 5 |
| 6. Gaan uw werkzaamheden u beter af dan voor de operatie? | 1 | 2 | 3 | 4 | 5 |
| 7. Heeft de operatie een uitkomst geboden voor de klachten die u had? | 1 | 2 | 3 | 4 | 5 |
| 8. Hoe tevreden bent u over het resultaat van de operatie? | 1 | 2 | 3 | 4 | 5 |
| 9. Zou u opnieuw deze operatie ondergaan indien u dezelfde klachten weer zou hebben? | 1 | 2 | 3 | 4 | 5 |
| 10. Zou u deze operatie iemand anders aanraden? | 1 | 2 | 3 | 4 | 5 |
| 11. Maakt u vanwege uw knie nog gebruik van fysiotherapie? | 1 | 2 | 3 | 4 | 5 |
| 12. Heeft u uw werkzaamheden moeten aanpassen? | 1 | 2 | 3 | 4 | 5 |
