# Supplementary material for: Meniscal allograft transplantation in The Netherlands: long-term survival, patient-reported outcomes, and their association with preoperative complaints and interventions
Source: Knee Surg Sports Traumatol Arthrosc. 2020 Sep 26;28(11):3551–60. doi: 10.1007/s00167-020-06276-y (PMC7591451; doi:10.1007/s00167-020-06276-y)
Supplement: Supplementary file 2 — Supplementary material 2 (DOCX 13 kb) [file 167_2020_6276_MOESM2_ESM.docx]

**Appendix II**

Questions on satisfaction (in English)

|  | Not at all | Little | To a reasonable degree | Much | Very much |
| --- | --- | --- | --- | --- | --- |
| 1. Do the results of the operation meet your expectations? | 1 | 2 | 3 | 4 | 5 |
| 2. Did the operation make you more confident? | 1 | 2 | 3 | 4 | 5 |
| 3. Has the operation had a positive effect on your social life? | 1 | 2 | 3 | 4 | 5 |
| 4. Are you more satisfied with your body since the operation? | 1 | 2 | 3 | 4 | 5 |
| 5. Do your daily activities work better than before surgery? | 1 | 2 | 3 | 4 | 5 |
| 6. Are your work activities going better than before surgery? | 1 | 2 | 3 | 4 | 5 |
| 7. Has the operation provided a solution for the complaints you had? | 1 | 2 | 3 | 4 | 5 |
| 8. How satisfied are you with the outcome of the operation? | 1 | 2 | 3 | 4 | 5 |
| 9. Would you undergo this surgery again if you would have the same complaints? | 1 | 2 | 3 | 4 | 5 |
| 10. Would you recommend this operation to someone else? | 1 | 2 | 3 | 4 | 5 |
| 11. Do you have physical therapy because of your knee? | 1 | 2 | 3 | 4 | 5 |
| 12. Did you have to adjust your work? | 1 | 2 | 3 | 4 | 5 |
